# Lasting Impact of Patient‐Led Medical Education

**DOI:** 10.1111/tct.70147

**Published:** 2025-07-12

**Authors:** Bonita Sawatzky, Cathy Kline

**Affiliations:** ^1^ Department of Orthopaedics, Faculty of Medicine University of British Columbia Vancouver British Columbia Canada; ^2^ Patient & Community Partnership for Education, Office of UBC Health The University of British Columbia Vancouver British Columbia Canada; ^3^ P. A. Woodward Instructional Resources Centre (IRC) Vancouver British Columbia Canada

**Keywords:** case‐based assessment, health mentors, interprofessional education, medical education, patient's voice, shared decision‐making

## Abstract

**Background:**

Our university offers an interprofessional program to medical students in Year 1 of a 4‐year undergraduate medical program: Health professional students learn from a health mentor—someone living with a chronic condition. This helps foster patient‐centredness, empathy and communication skills. Long‐term assessment of patient involvement in medical education is rare; thus, this study explores the lasting effects of 3‐year post‐program at entry‐to‐practice.

**Methods:**

We conducted a case‐based study of fourth‐year medical students to evaluate the impact of learning from patients in the Health Mentors Program (HMP). Students analysed a video case of a person with cerebral palsy who fell at home and created a care plan. We compared students who participated in the HMP with those who did not, assessing how often they considered the patient's and caregiver's perspectives, the number of diagnostic tests ordered and referrals to other professionals and community services.

**Findings:**

*T*‐tests showed that HMP students significantly prioritised the patient's and caregiver's voices (*p* = 0.014, Cohen's *d* = 0.6) and ordered fewer diagnostic tests than non–HMP students (*p* = 0.001, Cohen's *d* = 3.3). However, there were no significant differences in medical consults, referrals to allied health professionals or community services.

**Conclusions:**

This was the first, limited attempt to use case‐based assessments to measure the long‐term impact of patient‐centred learning. Integrating patient perspectives into preclinical education may enhance students' ability to work collaboratively with patients in care planning. Designing structured assessments around patient‐centred care can help ensure that students retain and apply these skills in their clinical careers.

## 1 Introduction

The short‐term benefits of patient involvement for undergraduate students and patients are well documented. By patient involvement, we mean the active involvement of real patients who are experts by experience and educators of health professional students, not simulated patients or the clinical interactions students have in which they learn from patients. Immediate outcomes for learners include enhanced patient‐centredness, a deeper understanding of illness experiences, increased empathy and improved communication skills [1, 2, 3, 4]. Similarly, patients who participate in these educational programs report increased self‐efficacy, improved self‐esteem and a sense of fulfilment from contributing to patient‐centred care and the healthcare system [2, 4, 5, 6]. However, there is limited research on whether these benefits persist over time and ultimately translate into improved patient care.

In 2011, the University of British Columbia introduced the Health Mentors Program (HMP)—an elective initiative that engages patients as educators [5]. This program invites individuals with lived experiences of chronic illness and disability to facilitate small‐group learning for health professional groups of students from diverse disciplines, including medicine, dentistry, occupational therapy, physiotherapy, nursing and kinesiology (three to four per group). The program's primary objectives are to foster interprofessional collaboration, promote patient partnerships and enhance shared decision‐making (SDM) in managing chronic conditions.

Health mentors are ‘experts by experience’ in living with and managing chronic disease or disability. Students learn directly from the health mentors through an authentic patient‐centred model of education, in which the locus of learning is the student and mentor, with the expert professional teacher as a resource. It is an elective interprofessional education activity, and each program determines how it fits into their curriculum. In medicine, HMP is offered as an option in the first year of a 4‐year undergraduate medical program as part of the Flexible Enhanced Learning (FLEX) program that allows students to explore their learning goals in greater depth. Originally structured as a 16‐month program with six sessions, it has since been condensed into a shorter 9‐month format to better fit with curriculum structures (see Table 1).

**TABLE 1:** Format of the Health Mentors Program.

Date	Topic	Sample discussion questions
September	Meeting 1: Orientation and Introduction to the Health Care Team (3 h)	Members of the Healthcare Team: We suggest you go around the group, each student describing the role their profession plays in healthcare and why you chose this particular profession. You should also describe how your educational program prepares you to play this role. What values distinguish your profession from others? How are your professions represented on the mentor's healthcare team?
October	Meeting 2: Words and Meanings, and Why They Matter (2 h)	(a) Discuss the following clusters of words and add other related terms if you wish: health, disease, illness and disability… What do these words mean to you? Patient, client, consumer, service user and survivor… What words do you use in your professional program and why? Normality, recovery, coping and self‐management … (b) What do we mean by quality of life? (c) Discuss your experiences of the following: stereotypes, stigma, cultural differences and generational differences.
November	Meeting 3: Living with Chronic Disease/Disability and Its Management (2 h)	The mentor should decide whether to start with their life story first, and illness journey second, or vice versa. If it is not possible to cleanly separate the two, decide how best to present both these aspects of their lives in parallel. The group should decide how they would like to capture the information (e.g., construct a chronology).
January	Meeting 4: The Healthcare Team and Patient/Client‐Centred Care (2 h)	The Mentor's Care Team: There will be many individuals and organisations involved in the health mentor's care. The mentor may or may not have a formal care plan. The group should try to map out as a diagram who is involved in the mentor's care: indicate who is working as a team and who may not know of each other's existence. If the mentor does not have a care team, the group should try to map out what might be an ideal care team.
February	Meeting 5: Finding, Managing and Sharing Health Information (2 h)	Ask the mentor to talk about their experiences of health information. For example, what information did they need and get at different stages in their chronic condition? How did their needs change and why? What different sources of information were available? What was most useful and informative? What sources of health information are available, including community sources such as peer support? How do you, as students, health professionals or patient/clients, manage information overload? How do you assess the quality of health information? What is ‘good evidence’?
March	Optional meeting to plan the symposium (3 h)	The purpose of this meeting is to discuss and summarise what both students and mentor have learned so far and agree on what you would like to share with the other groups. (The symposium is attended by students and mentors.) Each group will agree on one key piece of learning (i.e., the most important idea or theme that you want to share with other groups) and write this in the form of a Twitter tweet.
April	Meeting 6: Symposium (3 h)	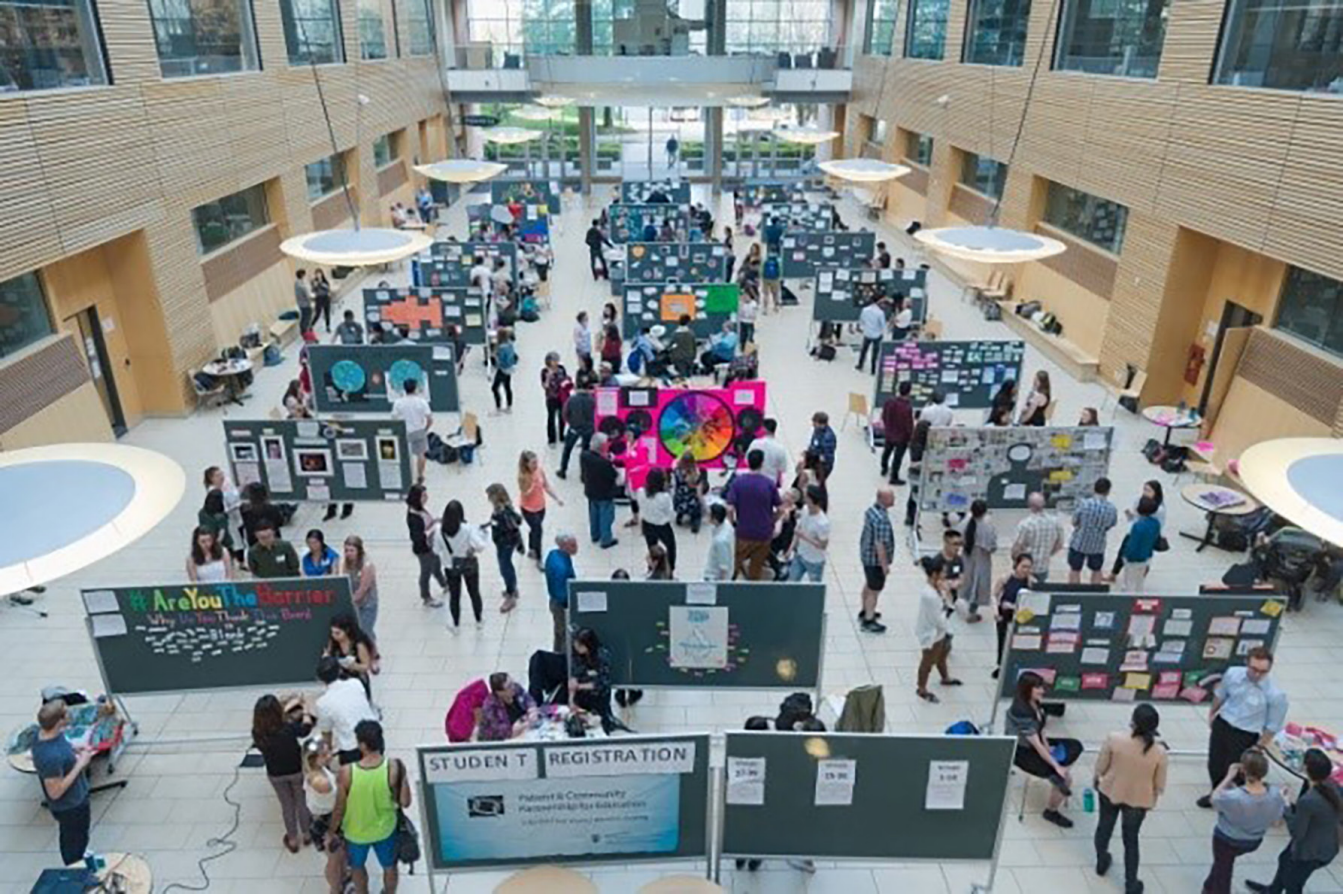
May	Meeting 7: Partnerships, Collaboration, Shared Decision‐Making and the Future (2 h)	What have you learned about shared decision‐making in the process? What have you learned about decision‐making in healthcare through your clinical experiences? What do you think about concepts of ‘compliance’ or ‘adherence’? Identify the key principles that will be relevant to shared decision‐making in the healthcare setting.

To evaluate the program's effectiveness, we tracked initial learner outcomes using student reflective journals, program surveys (including rating scales and open‐ended responses), focus groups and interviews. These assessments have consistently demonstrated high satisfaction levels (4.1/5) and transformative learning experiences aligned with the program's goals [5]. This study investigated whether these outcomes might endure through the students' undergraduate training and influence their clinical practice.

Assessing long‐term outcomes in medical education presents significant challenges. It requires tracking learners from medical school through residency and into clinical practice to determine how early experiences shape patient care [7]. Evaluating the impact of a specific preclinical educational innovation is further complicated by dilution effects [8] and the inherent difficulty of measuring clinical reasoning and decision‐making skills [9]. While the objective structured clinical examination (OSCE) is a valid assessment tool, it is logistically complex and costly to implement across all curricular innovations.

Using an innovative standardised case‐based assessment may offer a more practical, cost‐effective alternative, allowing evaluation of clinical competencies both in‐person and online—aligning with the shift towards remote learning following the pandemic. Like the OSCE, case‐based assessments can be designed to measure clinical skills related to specific learning objectives.

Therefore, the first objective of this study was to assess the long‐term impact of the HMP by comparing whether participating graduating students were more likely to incorporate the patient's voice in their assessments and care plans versus non–HMP students and whether participation influenced other clinical decision‐making aspects, such as the number and type of diagnostic tests or referrals made to healthcare professionals and community services that differed between the two groups. The second objective was to evaluate the feasibility of using a more standardised case‐based assessment to detect differences between students who participated in the program and those who did not.

## 2 Materials and Methods

This was a quantitative study that used a case‐based assessment to compare the difference in students who participated in the HMP compared to those who did not. We chose this approach because case studies depict real‐life problems to solve that are amenable to objective scoring and are commonly used to contextualise teaching, learning and assessment. Although cases are often written, we chose to use a video depicting a clinical scenario that reflected common issues related to living with a chronic condition, making it both engaging and relevant. Ethical approval for this study was granted by the UBC Behavioural Research Ethics Board.

### 2.1 Participants

Fourth‐year medical students were eligible for inclusion in this study from the UBC Vancouver learning site. Participation in this study was voluntary, and students were told that the aim of this study was ‘*to evaluate how different course selections during medical training affect how students prioritize health care treatment options’* with no specific reference to the HMP, to minimise bias. Students who consented to have their care plan included in this study submitted it electronically to the researchers. The assessment was done for 2 years. In Year 1, it was an option during class time (*N* = 80). Students were given 20 min to write the care plan during class time. This activity was followed by patient‐led discussions with the whole class. In the second year, it was an option to participate in this study, outside of class time (*N* = 23).

### 2.2 What We Did

The case‐based assessment was administered at the end of the final term of a 4‐year medical school program before they dispersed for residency training. We used a video developed by a team of disability researchers and patients from Monash University, Australia [10]. Students were provided a link to watch a video for the assessment. In the video, Sara, who has cerebral palsy, has a fall, is found by a care aid and is taken to the local hospital emergency department by ambulance. Students were asked to write a care plan as the attending physician. There were no direct questions about the HMP.

**FIGURE 1:**
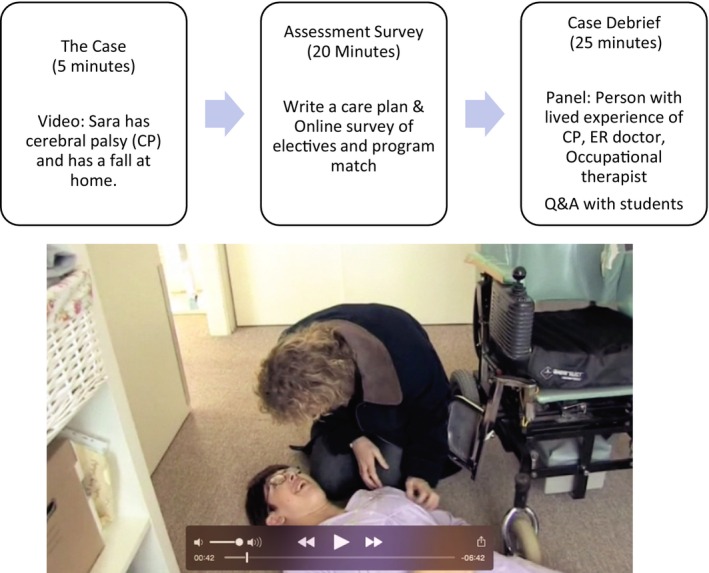
The case‐based assessment process embedded in a ‘Preparation for Medical Practice’ class in students' 4th year.

To include this assessment as a learning opportunity, after students submitted their care plan, we debriefed the case with a panel of experts, including an emergency department doctor, occupational therapist and a patient living with cerebral palsy. Each panel member discussed how they would approach the situation and answered questions from students. The panellists' role was to discuss the case with the class. This discussion was not part of the assessment nor used for scoring. Figure 1 outlines the process.

We collected basic demographic data including student's age and gender, their care plan and what program students were matched to for their residency. Care plans were compared using variables such as the patient/caregiver voice in their plan, the number of diagnostic tests, the number of allied health professional referrals, the total number of consults and the number of community services. Students completed it using an online portal, Qualtrics (Version 2015) with no identifying data collected. However, we did ask for their student's ID, but this was only used to determine if they had participated in the HMP or not.

### 2.3 Analysis

Each student's care plan was analysed by two investigators blinded to the student's group. Students were given one point for each reference to the (1) patient/family perspective, (2) number of diagnostic tests, (3) number of allied health professional referrals, (4) number of community service referrals made and (5) total number of consults (see Table 2). In this example, ‘with collateral from mother’ and ‘make sure to discuss with Sara’ would each receive a point for a total score of two references to the patient/family perspective. Levene's *T*‐tests were used to test the count differences for these variables between the two groups (HMP vs. non‐HMP), because the data were not normally distributed and there were uneven group sizes. Significance was set at *p* < 0.05. To assess the effect size, Cohen's *d* was used. A small effect size is typically considered to be around 0.2, medium effect size is around 0.5 and large effect size is around 0.8.

**TABLE 2:** Example of care plan scoring.

Care plan	Patient/caregiver input	Allied Health referrals	Medical consults	Medical tests ordered	Community service reviews/referrals
Full history and physical with collateral from mother and home support working. Make sure to discuss with Sara. I would do basic screening bloodwork, an ECG, and likely do a CT head to rule out reasons for a fall and a haemorrhage. Also, I would order anti‐seizure med levels. She should see an OT regarding ADLs and IADL management. Because she is home alone, we need to figure out if she is safe to go home or if there are other resources she needs, so we can support her independence. She should see a physio to assess her transfers and to maximise her mobility. SLP to see regarding maximising communication ability. Neuro to assess current medications to see if they may be contributing to a fall or to order an EEG if necessary. [HMP graduate, Internal Medicine Resident]	2	3	1	4	1
Initially, she would receive bloodwork: CBC, lytes, bun, cr, LFTs, TSH, epilepsy medication levels as general assessment for underlying condition that may have caused the fall, X‐ray of shoulder, consult neurology (+/− head CT) due to unwitnessed fall and should be seen by PT, OT and social worker to assess safety of home environment. [Non–HMP graduate, Internal Medicine Resident]	0	3	1	8	0

### 2.4 Results

Sixteen of the 71 HMP students (response rate 23%) and 87 of the 401 non–HMP students (response rate 22%) submitted care plans for this study. HMP and non–HMP students had similar characteristics with respect to age and gender (Table 3).

**TABLE 3:** Student characteristics by group.

	HMP% (*N* = 16)	Non‐HMP% (*N* = 87)
Mean age (yrs)	28	27
Females	62.5	59.8
Males	37.5	40.2
Other gender	0.0	0.0
Residency/Specialty Program		
Anaesthesiology	0.0	3.4
Emergency Medicine	0.0	4.6
Family Medicine	18.8	35.6
Internal Medicine	37.5	13.8
Neurology	6.3	3.4
Obstetrics and Gynaecology	0.0	3.4
Ophthalmology	0.0	2.3
Pathology	6.3	1.1
Paediatrics	6.3	5.7
Physiatry	0.0	1.1
Physical Medicine and Rehabilitation	0.0	2.3
Psychiatry	18.8	4.6
Public Health and Preventative Medicine	0.0	1.1
Radiology	6.3	2.3
Surgery	0.0	9.2
Unmatched or no response	0.0	5.7

Five of the 16 HMP students (31%) while only 15 of the 87 non–HMP students (17%) made at least one reference to involving Sara or her caregivers. The *T*‐test showed that the HMP group made significantly more statements that included the patient (*p* = 0.014, Cohen's *d* = 0.6). HMP students also ordered significantly fewer diagnostic tests than non–HMP students (*p* = 0.001, Cohen's *d* = 3.3). There were no significant differences between HMP students and non–HMP students in the number of medical consults, referrals to allied professionals or community services ordered. See Table 4 for comparisons.

**TABLE 4:** Mean number of references to variable per case plan.

Variable	HMP	Non‐HMP
Allied HCP referrals	2.31	2.50
Medical consults	0.88	0.80
Community‐based referrals	0.44	0.35
*Sara/caregiver involvement	**0.43**	**0.23**
*Dx tests	**3.13**	**4.75**

* significant difference between groups (*p* < 0.05).

### 2.5 Discussion

Our study adds to the literature in two main ways. It offers a tested model to quantitatively assess the long‐term effects of patient involvement in health professional education that can be administered as part of a learning activity or as a separate assessment. Together with a previously published qualitative study of the long‐term effects of the HMP showing the program that fosters the development of a professional identity that embraces patient partnership and collaboration [11], the present study adds to the evidence that early exposure to patients as educators during preclinical training primes learners to see patients as capable of collaborating in the development of their care plan with expertise to bring to the relationship from their lived experience.

‘Patients as educators during preclinical training primes learners to see patients as capable of collaborating in the development of their care plan’.

The results of our study suggested that the HMP had a positive effect on medical students' intentions to involve patients in decision‐making at entry‐to‐practice. Including the patient in care planning decreased the number of diagnostic tests ordered. We surmised that students who demonstrated SDM were more confident in good communication with the patient, enabling proper diagnosis and treatment, and thus were less reliant on medical testing that would be more taxing to the patient and healthcare system. Many non–HMP students wrote significantly more lists of medical tests and consults that resembled a script for making a differential diagnosis—the discourse most rewarded in clinical settings. The HMP may have had an inoculating effect against a hidden curriculum in which traditional bedside evaluation tools may receive less attention because of the technological advances of diagnostic imaging and laboratory testing [12]. As noted in Table 3, more HMP students were entering residencies/specialties post‐graduation that allowed them to spend more time with patients, such as internal medicine and psychiatry. These findings align with a previously published qualitative study that explored professional identity formation. In that study, the HMP influenced their decision towards a residency program that would allow them to spend more quality time with patients [11].

‘Including the patient in care planning decreased the number of diagnostic tests ordered’.

Notably, contemporary research suggests that patient narratives, when integrated into medical education, enhance students' perceptions of a SDM approach, which is one of the goals of our HMP. Eggeling et al. [13] used a measure in their study to ask specifically about how much influence the patient should have when making decisions on their healthcare. Their questionnaire included topics about the perceived importance of SDM, attitudes towards the participation of patients with Parkinson's disease (PD) and attitudes towards SDM in situations where more than one treatment option was available. They compared learners' responses (drop down menu of choices) to a reading of a patient's narrative versus a fact‐based information document about a patient with PD. They found that those who read the narrative were more likely to have an SDM approach by involving the patient more in the process. However, there was no difference in the final decision for treatment. In contrast, our study was more open‐ended in the assessment, with our attempt as researchers to quantify by specific word references only. Both methods have their limitations, and we acknowledge that written references to the patient's perspective in care plans remain only a proxy indicator for patient‐centred care. Longitudinal studies are necessary to assess the lasting influence of preclinical patient involvement on professional practice.

From an interprofessional learning perspective, referrals to allied health professionals were generally appropriate for the case in both groups, with the mean number of references to community services slightly higher in the HMP group but non‐significant. We expected to see more references to community services in these students' care plans, given the emphasis on learning about the value of community resources and peer supports in the HMP. In the CanMEDS competency framework used by Canadian medical schools, the role of ‘Medical Expert’ is the central role of physicians and defines the physician's scope of clinical practice [14]. The centrality of the ‘Medical Expert’ role may inadvertently narrow students' focus on this competency at the expense of non‐medical community‐based resources, particularly in an assessment situation. If there were increased collaboration with community services and peer supports as a core part of interprofessional education, embedded in curriculum, we may see increased community choices being offered to patients as standard care.

The lack of learning about the value of community resources and peer supports in health professional education led us to co‐develop with health mentors and students a database of learning resources, recommended by patient and community organisations and evaluated by students [15]. Many health mentors had been sharing resources with their groups since the program began, and we wanted to make these resources available to all students. To keep the database up to date and to reinforce the importance of community resources in chronic disease management, we added it as an activity to HMP Session #5 on finding, managing and sharing health information. The groups are now asked to identify an online resource that is useful for both mentors and students and submit it for inclusion in the database.

## 3 Limitations

This study is not without limitations. The results are based on a case‐based assessment without any direct evidence of student behaviour in practice. The program is also limited in the number of students who can participate each year, and therefore there are far more medical students who do not take the program. We acknowledge that the unbalanced sample sizes between groups can impact estimates of effect size. As students approach graduation, their focus shifts towards licensing exams, which likely contributed to the low response rate—particularly in the second year when the case‐based assessment was conducted outside of class time. Additionally, selection bias may have influenced the results: Students who choose a program that includes patient mentorship may already be inclined towards SDM. However, students register for the HMP during the first week of classes when the sheer number of available opportunities can be overwhelming, potentially leading some to miss or misunderstand the program's purpose. Notably, many students later express regret at not enrolling after registration closes, suggesting that selection bias may not fully account for the findings. We do not know what other factors in their personal or professional lives might have influenced the results or if the differences observed will be maintained over time. Even at graduation from medical school, the students are still early in their careers, and it is likely that their residency programs will strongly shape the way they engage with patients in practice.

The results of this study are based on a single case assessment as representative of what students learned, which is a limitation. It is truly difficult to assess such programs; thus, much of our work in the past has been qualitative in nature [6, 11] when the sample sizes are greatly reduced. This study attempted to reach a larger group of students and to compare two groups of learners. In the future, one approach would be to develop a longitudinal study over the 4 years whereby students completed case‐based assessments including reflective responses that were repeated at the beginning of each year to plot the trajectory of change in their own answers over the 4 years. We could compare within the students' learning and between groups depending upon program initiatives that focused on interprofessional and/or patient‐led education. A longitudinal approach would give the methods more robustness and ability to scale.

The informal nature of the evaluation is also a limitation. The case‐based assessment has no real interaction with the simulation patient, unlike an OSCE, but it does provide context for the student to explore the patient's life. In addition, as limited to one case, we cannot assume that future doctors who did not go through the HMP will not include the patients' voices in their subsequent practice. They are still early in their training, and their residency will have significant influence on their future practice styles. Future research into their behaviour with patients in clinical settings or thematic analysis of students' reflections could enhance the credibility of the results.

Despite these limitations, this study highlights the potential of case‐based assessments as a practical and cost‐effective method for evaluating patient‐centred learning experiences, such as the HMP, where patients serve as educators. By clearly defining assessment criteria and selecting cases aligned with specific learning objectives, educators can better measure the impact of innovative learning methods like the HMP in undergraduate health professional education to assess the potential longer term effects.

‘The study highlights the potential of case‐based assessments as a practical and cost‐effective method for evaluating patient‐centered learning experiences’.

## 4 Conclusions

Preclinical learning from patients as mentors can improve SDM and reduce unnecessary biomedical testing in care planning at entry‐to‐practice and potentially influence students' career choice. Case‐based assessment may be a method to measure the long‐term impact of patient‐centred learning, particularly when it is designed and implemented as a classroom learning activity. Learning about patient‐centred care directly from patients in the preclinical curriculum can prepare learners to work in partnership with patients in practice.

## Author Contributions

**Bonita Sawatzky:** conceptualisation, investigation, formal analysis, methodology, supervision, writing — original and reviewing/editing. **Cathy Kline:** conceptualisation, data curation, investigation, project administration, methodology, supervision, writing — original and reviewing/editing.

## Ethics Statement

Ethical approval for this research was granted by the UBC Behavioural Ethics Review Board (H15‐00102).

## Conflicts of Interest

The authors declare no conflicts of interest.

## Data Availability

Anonymised data may be made available upon request.
